# Design and Characterization of Carboplatin and Paclitaxel Loaded PCL Filaments for 3D Printed Controlled Release Intrauterine Implants

**DOI:** 10.3390/pharmaceutics15041154

**Published:** 2023-04-05

**Authors:** Cem Varan, Davut Aksüt, Murat Şen, Erem Bilensoy

**Affiliations:** 1 Department of Pharmaceutical Technology, Faculty of Pharmacy, Hacettepe University, Ankara 06100, Turkey; 2 Department of Chemistry, Faculty of Science, Hacettepe University, Ankara 06800, Turkey; 3 Polymer Science and Technology Division, Institute of Science Hacettepe University, Beytepe, Ankara 06800, Turkey

**Keywords:** 3D printer, carboplatin, cyclodextrin, intrauterine device, polycaprolactone, paclitaxel, personalized drug, uterine cancer

## Abstract

Uterine cancer is the fourth most common cancer in women. Despite various chemotherapy approaches, the desired effect has not yet been achieved. The main reason is each patient responds differently to standard treatment protocols. The production of personalized drugs and/or drug-loaded implants is not possible in today’s pharmaceutical industry; 3D printers allow for the rapid and flexible preparation of personalized drug-loaded implants. However, the key point is the preparation of drug-loaded working material such as filament for 3D printers. In this study, two different anticancer (paclitaxel, carboplatin) drug-loaded PCL filaments with a 1.75 mm diameter were prepared with a hot-melt extruder. To optimize the filament for a 3D printer, different PCL M_n_, cyclodextrins and different formulation parameters were tried, and a series of characterization studies of filaments were conducted. The encapsulation efficiency, drug release profile and in vitro cell culture studies have shown that 85% of loaded drugs retain their effectiveness, provide a controlled release for 10 days and cause a decrease in cell viability of over 60%. In conclusion, it is possible to prepare optimum dual anticancer drug-loaded filaments for FDM 3D printers. Drug-eluting personalized intra-uterine devices can be designed for the treatment of uterine cancer by using these filaments.

## 1. Introduction

Uterine cancer, including endometrial cancer and uterine sarcoma, is characterized by the uncontrolled growth and cancerization of cells in the uterus. According to the 2022 statistics of the American Cancer Society, uterine cancer is the fourth most common and sixth most deadly cancer in women [1]. Although the incidence and mortality rates in most cancer types decrease with early diagnosis and new treatment approaches, this is not the case in uterine cancer and mortality increases over the years [2].

The most preferred treatment option is the surgical removal of the cancerous area. Following surgery, adjuvant therapy is applied to uterine cancer patients [3]. Although surgery is more successful than other treatment options, it directly affects fertility and can cause a serious problem, especially for patients who have never had children [4]. Therefore, it is very important to develop an effective and safe chemotherapeutic approach that can be an alternative to surgery. Various chemotherapy drugs and their combinations are known to be effective in the treatment of uterine cancer [5,6,7]. Especially in endometrial cancer, paclitaxel (PCX), carboplatin (CBP) and the combinations are used in clinics [8]. However, an effective chemotherapy approach that can be a complete alternative to surgery has not been put forward. The main reason is that each patient’s response to treatment is different, and the therapeutic dose or combination varies for each patient. Although today’s pharmaceutical industry produces standard doses of drugs for each patient, there is a need to produce personalized drugs for each patient for successful cancer treatment; 3D printing of drug-loaded implants offers hope for overcoming this limitation; 3D printers allow the production of pharmaceutical dosage forms or implants by personalizing many parameters such as shape, size, geometry, and drug dose [9,10,11]. 

In addition, the uterus is a very accessible organ for the application of local drug delivery systems such as a drug-loaded intrauterine device (IUD) [12]. A personalized drug-loaded IUD can be applied directly to the cancerous area in the treatment of uterine cancer. Thus, the patient will not be exposed to possible side effects and problems such as rapid elimination in the systemic circulation of the drug. IUDs prepared with 3D printers are very promising for the personalized treatment of uterine cancer [13,14].

The most important factor limiting the use of 3D printers in the pharmaceutical industry is a lack of materials containing stable and precisely dosed drugs. Nowadays, if the appropriate filaments are prepared to allow printing, it will be possible to prepare personalized implants (e.g., IUD) or drugs easily. Especially polymers such as polylactic acid and polycaprolactone (PCL), which have been used safely in biomedical products for many years, promise hope [15]. 

PCL is a synthetic polymer with a semi-crystalline structure. The increase in molecular weight leads to a decrease in crystalline properties [15]. PCL is suitable for preparing filaments for printing with FDM (fused deposition modeling) 3D printers and provides working with low temperatures (around 60–100 °C). In addition, it is a biodegradable and biocompatible polymer [16,17]. It has also been used safely in the medical field for many years as an FDA-approved polymer. Although PCL allows printing at low temperatures, these temperatures can still affect the stability of anticancer drugs. Therefore, even if the filament is prepared and printed at low temperatures, it is necessary to take additional precautions to ensure the stability of anticancer drugs. In addition, another important problem of anticancer drugs such as PCX is low solubility in water. Even if this drug is successfully loaded into filaments, it will need to dissolve in water in the application area and reach the cells. Hence, increasing the solubility of the drug is at least as important as ensuring its stability. Nanoparticle carriers have an important effect on increasing the solubility of drugs and maintaining their stability. Cyclodextrins are natural polymers that can carry drug molecules in their inner cavities thanks to their unique structure. They form the inclusion complexes with these properties to increase the solubility of the drug as well as protect it from external effects and increase its stability [18,19,20]. They are accepted as “generally recognized as safe” (GRAS) and have been used as pharmaceutical excipients for many years [21,22]. 

The aim of this study is the optimization and characterization of anticancer drug containing PCL filaments that will allow the 3D printing of personalized IUDs for the treatment of uterine cancer. For this purpose, two different anticancer drugs PCX and CBP with known efficacy on uterine cancer were selected as model drugs. Drug:CD inclusion complexes have been optimized to preserve the stability and solubility of drugs by using three different CD derivatives (hydroxypropyl-β-CD (HpβCD), methyl-β-CD (MβCD), and hydroxypropyl-γ-CD (HpγCD)) and two different anticancer drugs (PCX and CBP). Then, a series of characterization studies were carried out with three different PCL derivatives to select the most suitable for the preparation of filaments with a diameter of 1.75 mm. Filaments were prepared by using a hot melt extruder with these optimized PCL and drug:CD inclusion complexes. Finally, characterization studies, drug loading efficiency, drug release profiles and in vitro safety and anticancer effect studies were carried out. With this study, drug-loaded filaments that will allow the preparation of personalized IUDs or drugs to be used in cancer treatment, with a focus on uterine cancer, have been successfully prepared. Thus, a promising step has been taken to enable the preparation of personalized IUDs and implants manufactured through 3D printing.

## 2. Materials and Methods

### 2.1. Materials

CD derivatives (HpβCD, MβCD and HpγCD) were purchased from Wacker Chemie AG (Burghausen, Germany). PCX (>99.5%) obtained from LC Laboratories (Woburn, MA, USA). PCL derivatives (M_n_ = 10,000; M_n_ = 45,000 and M_n_ = 80,000), CBP (>99%) and all other chemicals were purchased from Sigma-Aldrich (Steinheim, Germany). Ultrapure water was obtained from Simplicity^®^ 185 Ultrapure Water System (Millipore, Molsheim, France).

### 2.2. Methods

#### 2.2.1. Preparation and Characterization of Drug:CD Inclusion Complex

Drug:CD inclusion complexes were prepared in 1:1 and 1:2 (drug:CD) molar ratios according to the co-precipitation method [19,22,23]. Briefly, 0.3% (*w/v*) drug was dissolved in ethanol and the amount corresponding to 1:1 or 1:2 molar ratios CD was weighed and dissolved in ultrapure water. Then, the drug solution was transferred into CD solution under a magnetic stirrer at 550 rpm for 7 days. The organic solvent was evaporated using a rotary evaporator and insoluble drug molecules were removed from final dispersion by using a PTFE syringe filter (0.45 µm). 

A series of characterization studies were carried out to show the formation of drug:CD inclusion complexes. First, the thermal properties of inclusion complexes were compared with free CD and drugs by using differential scanning calorimetry (DSC) and then the infrared bands were compared by using Fourier transform infrared (FTIR). DSC analyses were performed with Pyris Diamond (Perkin Elmer, Waltham, MA, USA) at 10 °C/min in the range of 25–250 °C for PCX and 30–280 °C for CBP under a nitrogen atmosphere. FTIR spectra were taken in the range of 4000–400 cm^−1^ using Spectrum Two (Perkin Elmer, Waltham, MA, USA) and attenuated total reflectance (ATR) techniques. 

Free PCX is found in the form of characteristic needle-like PCX crystals [24,25]. Scanning electron microscopy (SEM) images of inclusion complexes and free PCX were compared to show that the inclusion complex was successful, and PCX was in amorphous form. For this purpose, powdered inclusion complexes and PCX were coated with gold-palladium alloy with a thickness of 100 Å and imaged by using Nova NanoSEM 450 (FEI, Hillsboro, OR, USA). 

The encapsulation efficiency of the complexes was measured to determine the most suitable inclusion complex for further studies. Each inclusion complex was dissolved in acetonitrile for PCX and in ultrapure water for CBP, and then quantification was performed for each drug by validated HPLC methods. Equation (1) was used in the calculation of encapsulation efficiency.

(1) $$\mathrm{Encapsulation}\;\mathrm{Efficiency}\;\left(\%\right)=\frac{\mathrm{Measured}\;\mathrm{Drug}\;\mathrm{Amount}\;}{\mathrm{Initial}\;\mathrm{Drug}\;\mathrm{Amount}}\;\times \;100\;$$

#### 2.2.2. Characterization of PCL Polymers

Different characterization studies were carried out to determine the most suitable PCL type for preparing filaments with a diameter of 1.75 mm that will allow printing by using an FDM 3D printer. For this purpose, hardness analysis, thermogravimetric analysis (TGA), DSC analysis and melt flow index of three different PCL derivatives were examined. 

In the hardness analysis, the Shore D value of each polymer was measured with a Westop Type D durometer (Nishi Tokyo Seimitsu, Tokyo, Japan). Five measurement values from each serial sample were taken according to the Shore D hardness ASTM D 2240 standard. TGA analysis of polymers was performed under a nitrogen atmosphere with a heating rate of 10 °C/min and a flow rate of 20 mL/min in the range of 20–700 °C. DSC analysis was performed in the range of 25–250 °C as described in the previous section. MFI was performed with the Ceast Melt Flow Index (Instron, Norwood, MA, USA) at 75 °C under a weight of 1.2 kg.

#### 2.2.3. Preparation and Characterization of Filaments 

As a result of characterization studies, filaments with a diameter of 1.75 mm were prepared by using the hot melt extrusion technique [13,14,26] with optimal PCL and drug: CD inclusion complexes. For this purpose, PCL pellets and powder inclusion complexes were mixed and vortexed to contain a ratio of 1:1000 (w:w) PCX:PCL for PCX-loaded filament and 1:2000 (w:w) CBP:PCL for CBP-loaded filament. Each mixture was transferred to the feeder unit of the Composer 450 (3Devo, Netherlands). The heating zones of the device were set to 75, 80, 80 and 75 °C, respectively, and the screw speed was 2 rpm. 

The diameter of the filaments was measured against time by the optical sensor located at the filament output of Composer 450, and with the help of these data, the speed settings of the device were automatically optimized to remain at 1.75 (±0.1) of the diameter. In addition, these measurements were confirmed by re-measuring the different points of each filament by using a digital caliper (Scienceware^®^ Digi-Max, ABD, Wayne, NJ, USA).

To examine the effect of filament preparation on drugs or inclusion complexes, DSC and FTIR analyses described in Section 2.2.1 were performed by taking samples of 10 mg from each filament.

Stress-strain analysis was performed to determine the mechanical strength of drug-free or drug-loaded filaments. For this purpose, 7 cm samples (grip-to-grip distance is 5 cm) were taken from each filament and then were tested by using Universal Testing Machines Z010 equipped with 1 kN load cell (Zwick, Ulm, Germany) with pre-loaded 0.1 N and at 200 mm/min crosshead speed. All tests were repeated on three samples from the filament and average values were reported. [27,28].

Change in surface free energy of filaments was measured by contact angle analysis with three different liquids (paraffin, ethylene glycol and water). This liquid dropped on filaments and contact angles was measured by using a DSA100 Drop Shape Analyzer (Krüss, Hamburg, Germany). The surface free energy was calculated using the data in Young’s equation (Equation (2)) [28,29,30,31].

(2) $$\gamma_{L}\left(1+\cos\theta \right)=2\left(\sqrt{\gamma_{S}^{LW}\gamma_{L}^{LW}}+\sqrt{\gamma_{S}^{-}\gamma_{L}^{+}}+\sqrt{\gamma_{S}^{+}\gamma_{L}^{-}}\right)$$

Positron Annihilation Lifetime Spectroscopy (PALS) was used to detect nano-level gaps in the structure of filaments. ^22^NaCl was used as the positron source and the measurement was performed in the sandwich model. The resulting spectra were calculated according to Equation (3) using the LT9 program [28,29,31].

(3) $$\tau_{o-P_{s}}=0.5{\left[1-\frac{R}{R+∆R}+\frac{1}{2\pi }\sin\left(\frac{2\pi R}{R+∆R}\right)\right]}^{-1}$$

To calculate the theoretical drug amount in each filament, 1 cm samples were taken from each filament and weighed. Considering the % polymer, % CD and % drug ratios used in the preparation of the filament, the theoretical amount of the drug in the filament was calculated by dividing the drug weight by the total weight for each drug.

A 1 cm filament sample was dissolved in dichloromethane (DCM) (500 μL) to determine the amount of encapsulated drug. A 1 mL mobile phase used in the quantification of the drug for each drug was added and DCM was removed under the nitrogen atmosphere. Quantification was performed by validating the HPLC method for each drug; 1 cm samples were taken from each filament to determine the drug release profile of each filament. These samples were placed in ultrapure water, phosphate buffer (pH 7.4) and citrate buffer (pH 5.5) containing Tween-80 %0.1 (*v/v*). These samples are kept in a shaker water bath at 37 °C. The amount of released drugs was measured by HPLC at certain time intervals (1, 2, 5, 7 and 10 days).

#### 2.2.4. In Vitro cell Culture Studies 

L929 mouse fibroblast cell line which was defined as a standard method in USP and HEC-1B human endometrial adenocarcinoma cell line was used for the safety study of blank (drug-free) filaments. The anticancer effect of drug-containing filaments was examined on HEC-1B. 

L929 cell lines were cultured in Dulbecco’s modified Eagle’s medium (DMEM) (containing 10% fetal bovine serum, penicillin, and streptomycin) and HEC-1B cell lines were cultured in Eagle’s Minimum Essential Medium (EMEM) (containing 10% fetal bovine serum, penicillin and streptomycin). Both cell lines were cultured as a monolayer and maintained at 37 °C in a humidified 5% CO_2_ incubator. Cells were seeded in 12-well tissue culture plates and allowed to attach overnight. After 24 h, an empty ThinCert^®^ was placed in each of the wells to minimize the physical effect of filaments on cells. Formulations (filaments or drug solutions) were transferred into these ThinCert^®^ and incubated 72 h. At the end of the incubation period, the cell viability was determined by WST1 assay and optical densities (OD) were determined by a microplate reader (Molecular Devices, San Jose, CA, USA). Cell viability (%) was calculated as Equation (4).

(4) $$\mathrm{Cell}\;\mathrm{Viability}\;(\%)=\frac{\mathrm{Mean}\;\mathrm{Absorbance}\;\mathrm{of}\;\mathrm{Treated}\;\mathrm{Cells}}{\mathrm{Mean}\;\mathrm{Absorbance}\;\mathrm{of}\;\mathrm{non}-\mathrm{Treated}\;\mathrm{Cells}}\times 100$$

### 2.3. Statistical Analysis

All statistical analyses were performed using Student’s *t*-test by using GraphPad Prism 9 (San Diego, CA, USA). p < 0.05 was accepted as a statistically significant difference.

## 3. Results and Discussion

### 3.1. Preparation and Characterization of Drug:CD Inclusion Complex

**Figure 1:**
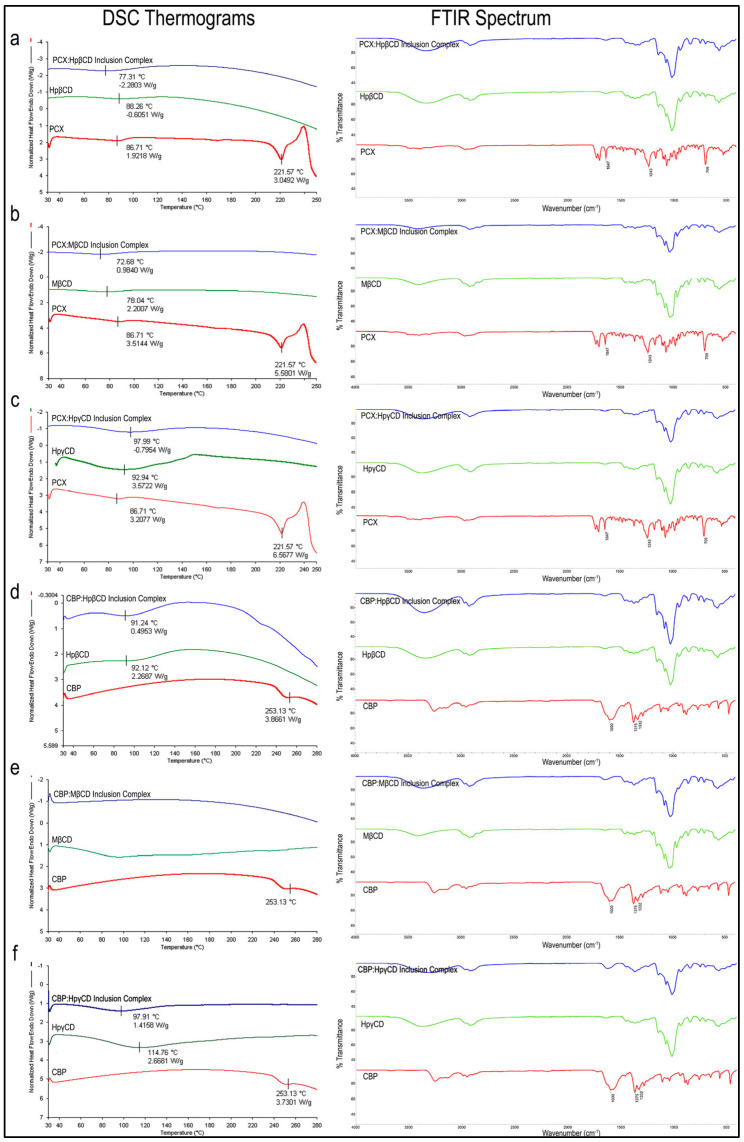
DSC thermograms and FTIR spectra of (**a**) PCX:HpβCD, (**b**) PCX:MβCD, (**c**) PCX:HpγCD, (**d**) CBP:HpβCD, (**e**) CBP:MβCD and (**f**) CBP:HpγCD inclusion complexes.

Inclusion complexes with three different CD derivatives at different molar rates were successfully prepared for both drugs. Figure 1 shows the DSC and FTIR analysis results of the inclusion complexes, free drug and CD.

#### 3.1.1. DSC Analysis

As seen in the DSC thermograms in Figure 1a–c, the endothermic peak of PCX was seen at 222 °C in accordance with the literature [32,33]. However, this melting peak was not present in inclusion complexes. This suggests that CD complexation changes the crystalline structure of the PCX by complexation into its internal cavity and maintaining the drug in amorphous form as no free crystals are observed in the thermogram. Similarly, the endothermic peak of CBP was also found at 253 °C, consistent with the literature [34,35] in Figure 1d–f. This peak was not observed in inclusion complexes and the thermal behavior of CBP within the inclusion complex was shown to change. In addition, an endothermic peak in the range of 80–100 °C was observed in both CD and inclusion complexes. All three CD derivatives are hydrophilic derivatives and have water absorption capacities. It is thought that these dehydration peaks are caused by the evaporation of water in the structure. 

#### 3.1.2. FTIR Analysis

FTIR spectra of PCX (Figure 1a–c) reveal the absorption band of the benzene ring in the structure seen at 1647 cm^−1^, C-N stress bands at 1243 cm^−1^ and aromatic group peaks at 709 cm^−1^ in accordance with the literature [32,36]. However, CD and PCX:CD inclusion complexes do not show these peaks, which confirms that inclusion complexes are formed, and free PCX is not present. 

Regarding CBP (Figure 1d–f), C=O vibration peaks at 1600 cm^−1^, Pt-NH_2_ at 1375 cm^−1^ and C-O at 1332 cm^−1^ are observed in accordance with the literature [37]. When the FTIR analysis of CD derivatives and inclusion complexes are examined, characteristic Pt-NH_2_ of the free CBP molecule is not present. Viewing the FTIR and DSC data together, it is seen that drug:CD inclusion complexes are successfully formed and there is no free drug molecule (PCX or CBP) for all formulations.

#### 3.1.3. SEM Images

**Figure 2:**
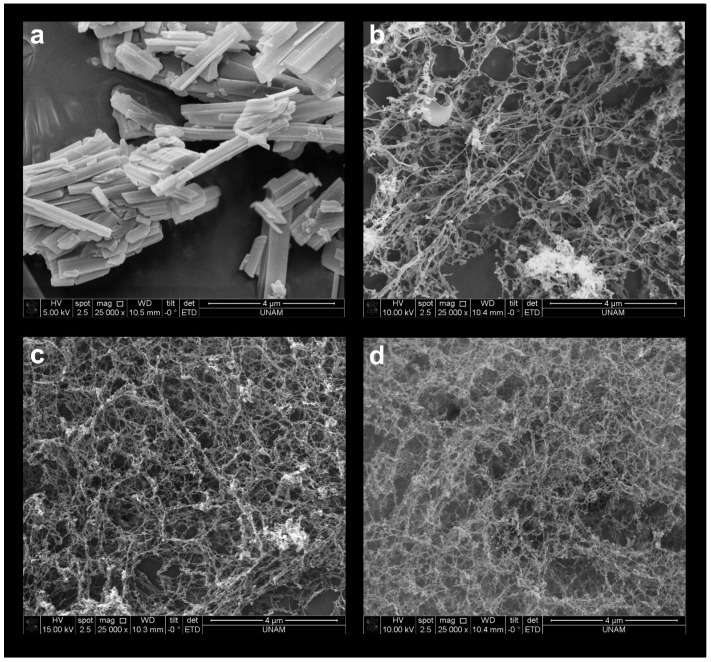
SEM images of (**a**) free PCX, (**b**) PCX:HpβCD, (**c**) PCX:MβCD and (**d**) PCX:HpγCD inclusion complexes.

When free PCX is examined under SEM, the characteristic sharp rods of PCX crystals are seen (Figure 2a) [24,25]. PCX:CD inclusion complexes were visualized with SEM to show that inclusion complexes are formed and that there is no free PCX. As seen in Figure 2b–d, PCX crystals were not seen in the inclusion complexes, although field scanning was performed. This is another indication that inclusion complexes are successfully formed and the free crystalline drug is not present.

#### 3.1.4. Encapsulation Efficiency

**Figure 3:**
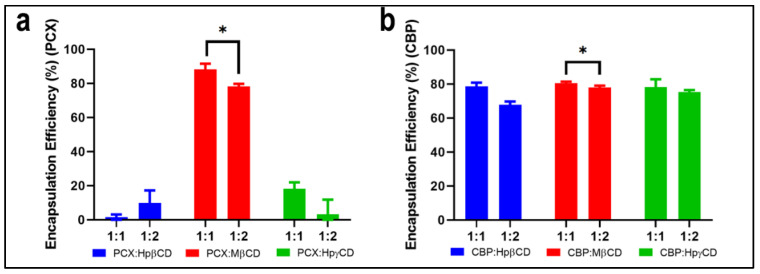
Encapsulation efficiency (%) of (**a**) PCX:CD (**b**) CBP:CD inclusion complexes (* the difference is significant, p < 0.05 (Student’s *t*-test), *n* = 3).

To determine which molar ratios and CD derivatives are optimal for the preparation of the inclusion complex, the encapsulation efficiency of each inclusion complex was measured and given in Figure 3. As can be seen, the highest encapsulation efficiency for PCX and CBP was achieved with MβCD inclusion complexes. 1:1 molar ratio drug:MβCD inclusion complex was found to be more effective in terms of drug loading than the 1:2 molar ratio complexes, and the statistical analysis showed that the encapsulation efficiency difference in the 1:1 to 1:2 drug:MβCD complexes was statistically significant (PCX: p = 0.0088, CBP: p = 0.044, *n* = 3).

### 3.2. Characterization of PCL Polymers

#### 3.2.1. Hardness and MFI Analysis

Hardness and MFI analysis of PCL with different molecular weights (PCL10 (M_n_ = 10,000), PCL45 (M_n_ = 45,000) and PCL80 (M_n_ = 80,000)) were performed and given in Table 1. Shore D values signifying the hardness of rigid materials are important in terms of showing the polymer’s resistance to thermal deformations. In the literature, PCL has been shown to have lower Shore D values than other polymers [38]. As shown in Table 1, an increase in the Shore D value in direct proportion to the increase in molecular weight is observed although the values are very close to each other. When the Shore D values were compared, it was seen that PCL10 was softer compared to other derivatives, while the hardest derivative was PCL80. According to this result, PCL80 can be considered more durable to deformation than other derivatives.

MFI is expressed as the value in grams of the polymer flowing in a molten state for 10 min under pressure and temperature [39]. This value is important to demonstrate the processability of the polymer. The MFI values obtained for each derivative are given in Table 1. The MFI of PCL10 is relatively high compared to that of PCL45 and PCL80 and has been found to be inversely proportional to molecular weight, consistent with the literature [40]. Such a high MFI value allows the polymer to flow quickly depending on the temperature. This rapid flow will make it quite challenging to adjust the filament diameter during the filament preparation process. PCL45 and PCL80 have lower MFI values than PCL10. The fact that the MFI value of PCL80 is lower than other derivatives, in accordance with the literature [41], shows that PCL80 will be advantageous in the controlled formation of filaments with a diameter of 1.75 mm by the hot melt extrusion process.

#### 3.2.2. TGA and DSC Analysis

**Figure 4:**
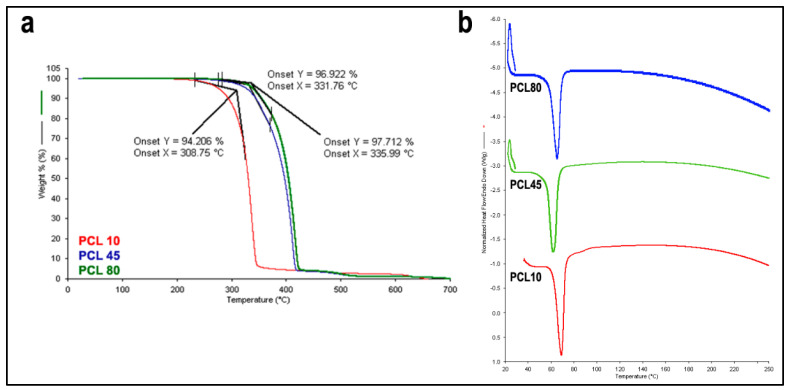
(**a**) TGA (**b**) DSC thermograms of PCL derivatives.

Thermograms of PCL10, PCL45 and PCL80 are given in Figure 4a obtained at the end of the thermogravimetric analyses by TGA. There is a single degradation peak in all PCL derivatives. It has been observed that PCL’s molecular weight and thermal resistance increase in direct proportion. PCL10 begins to degrade at 309 °C, while PCL45 at 332 °C and PCL80 at 336 °C. All PCL derivatives are completely degraded at 700 °C and the remaining ash is nearly zero. In line with the literature, the thermal resistance of PCL10 was found to be lower than that of the other PCL derivatives [42]. It was also observed that the difference between PCL45 and PCL80 was quite low. This is expected because the length of the molecular chain increases thermal resistance up to a certain point, but the effect on thermal resistance above a certain molecular weight remains limited like all physical properties except melt viscosity [43]. In the scope of these results, it can be said that PCL80 shows a higher thermal resistance than other PCL derivatives. 

DSC thermograms for PCL derivatives are given in Figure 4b. Endothermic melting peaks of all three derivatives were found to be quite close and were in the range of 60 °C to 70 °C in accordance with the literature [44,45,46]. According to these results, it is foreseen that all three derivatives can be extruded below 80 °C. 

In light of all these results, we decided to use the PCL80 derivative for the preparation of filaments. 

### 3.3. Preparation and Characterization of Filaments

FDM 3D printers are generally suitable for printing with filaments with a diameter of 1.75 mm or 2.85 mm, although they vary according to their model. However, FDM printers printing with filaments with a diameter of 1.75 mm are more widely available on the market [47]. For this reason, we aimed to prepare filaments with a diameter of 1.75 mm in this study. However, deviations of 0.1 mm in filament diameter are generally acceptable deviations for 3D printers, and filaments prepared in many studies have a margin of error of 0.1 mm [48,49]. 

**Figure 5:**
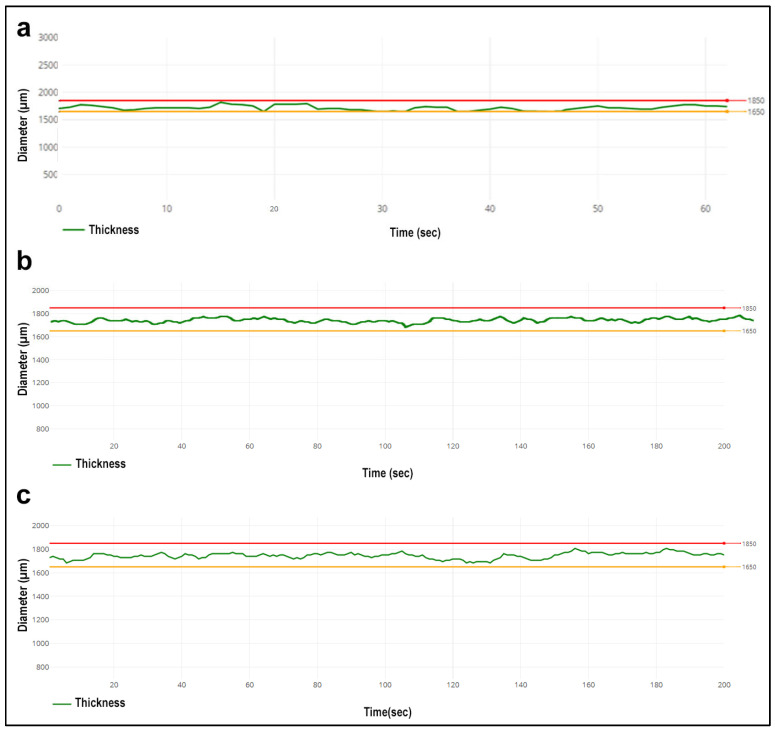
Time-dependent change of filament thickness during production (**a**) Drug-free PCL filament, (**b**) PCL filament containing PCX:CD inclusion complex, (**c**) PCL filament containing CBP:CD inclusion complex.

As described in Section 2.2.3, 3 different filaments have been prepared: PCX:CD-PCL, CBP:CD-PCL and blank PCL. The filament diameter of PCL filaments was made simultaneously during the extrusion process, and the filament’s diameter against production time was given in Figure 5. As can be seen, the polymer diameter varies between the lower limit of 1.65 mm and the upper limit of 1.85 mm. This indicates that the filaments are suitable for 3D printing in terms of thickness. In addition, measurements were made with the help of a digital caliper (Scienceware^®^ Digi-Max, Wayne, NJ, USA) from different parts of the filaments after extrusion. The obtained values were verified to be in the range of 1.85 mm to 1.65 mm. 

#### 3.3.1. DSC and FTIR Analysis

DSC analysis of blank and drug:CD loaded PCL filaments were performed to investigate changes in the melting peak of the PCL after the filament preparation process. 

**Figure 6:**
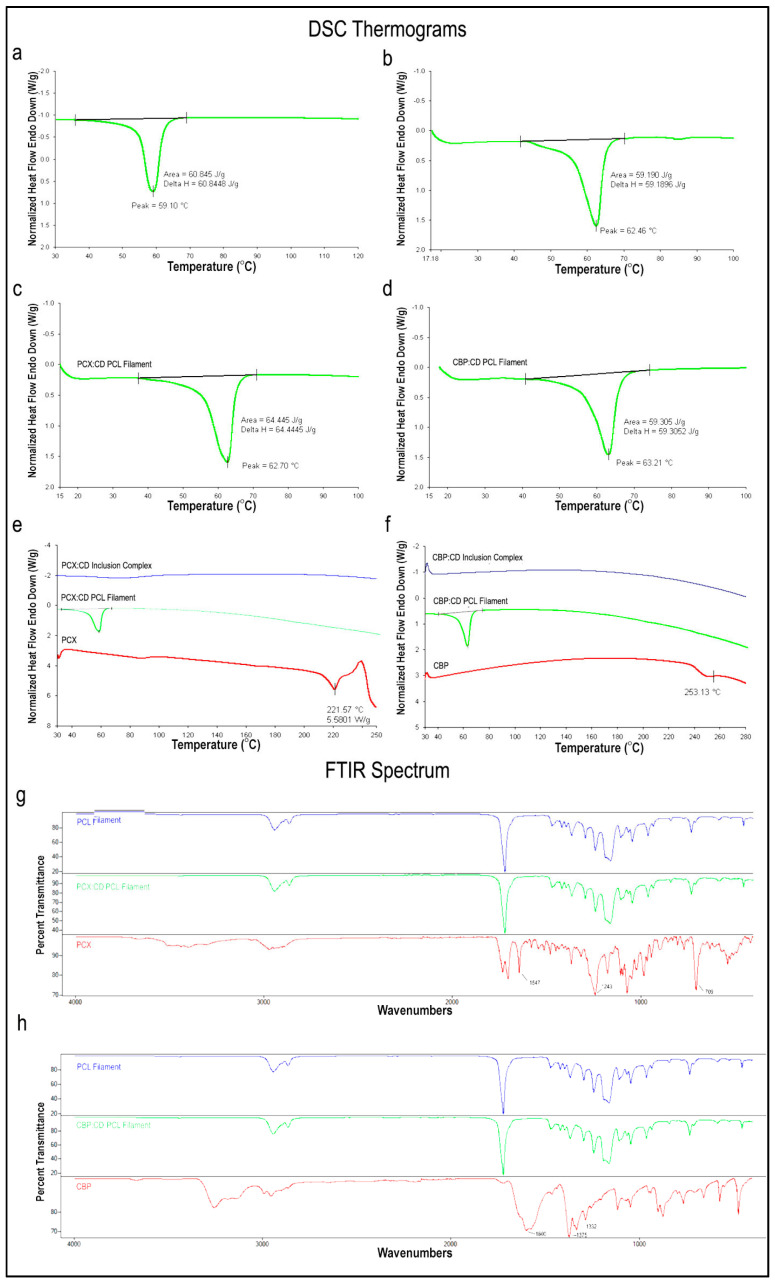
DSC thermogram of (**a**) PCL pellet, (**b**) Blank PCL Filament, (**c**–**e**) PCX:CD PCL Filament, (**d**–**f**) CBP:CD PCL Filament, and FTIR spectrums of (**g**) PCX:CD PCL Filament and (**h**) CBP:CD PCL Filament.

A comparison of blank and drug-loaded filament with PCL in pellet form is given in Figure 6a–d. The melting peak of the PCL (pellet) used in filament preparation was found to be very close to the melting peaks of blank and/or drug-loaded PCL filaments. Therefore, it can be said that the hot melt extrusion process does not affect the thermal properties of the PCL and thermal degradation does not occur during the process. 

We also investigated whether the free drug was released due to a possible degradation in the inclusion complexes after the preparation process. Each drug:CD inclusion complex loaded filament comparison of DSC thermograms of the related drug and inclusion complex is given in Figure 6e,f. As a result of the release of drugs with a degradation of the inclusion complex, endothermic peaks are expected to occur at 220–230 °C for PCX [32,33] and 230–260 °C for CBP [34,35]. However, this melting peak is not seen in the PCX:CD inclusion complex loaded filament. This suggests that CDs trap the PCX in its inner cavity and the drug did not release during or after the filament preparation stages. Similarly, CBP:CD inclusion complex loaded filament did not have a melting peak and it was shown that CBP remained within the inclusion complex during filament preparation.

When the FTIR spectrum of PCX is examined, the absorption band of the benzene ring (1647 cm^−1^), C-N stress peaks (1243 cm^−1^) and aromatic group peaks (709 cm^−1^) are seen in accordance with the literature [32,36]. However, PCX:CD inclusion complex loaded filaments do not show this band formation, which confirms that inclusion complexes retain their structure and free PCX is not present in the filament structure. When FTIR analyses of CBP:CD inclusion complex loaded PCL filaments are examined, the absorption band of Pt-NH_2_ (1375 cm^−1^) which is characteristic of CBP was not seen. FTIR and DSC data are taken together; it can be said that inclusion complexes maintain their structure during filament preparation and there is no free drug.

#### 3.3.2. Mechanical Properties

**Figure 7:**
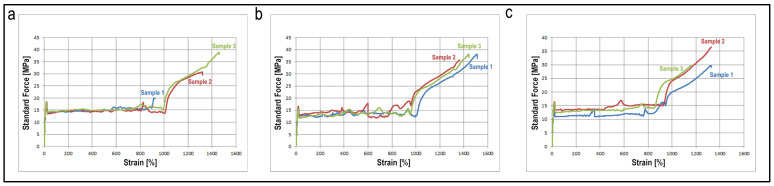
Stress/Strain graph of (**a**) Blank PCL Filament, (**b**) PCX:CD PCL Filament, (**c**) CBP:CD PCL Filament (*n* = 3, sample 1 (blue), sample 2 (red), sample 3 (green)).

The filaments’ mechanical properties directly affect the end product’s durability and provide information about 3D printability. It is essential to show whether the drug:CD inclusion complex added to the structure has a negative effect on the mechanical strength and flexibility of the PCL filament. As seen in Table 2 and Figure 7, the addition of CBP or PCX to the structure did not cause a statistically significant decrease in the elastic modules (E_mod_), stress at break (F_break_) and percent elongation at break (dL at break, %) of the PCL filaments, even increased stress at break. As seen in Figure 7b,c, and from the force values at 0.2% plastic strain (F at 0.2% plastic strain) given in Table 2 the addition of the drug:CD complex also increased the flexibility of the filament. Considering the mechanical properties of filaments studied for 3D printing in the study of Çevik et al., it can be said that all three filaments have appropriate strength and flexibility values for printing [50].

#### 3.3.3. Surface Energy

In order to determine the free surface energy of the filaments, three different liquids (paraffin, ethylene glycol and water) were used in accordance with the literature and contact angle analysis was performed. Based on the contact angle results, the surface free energy values were calculated with Young’s equation and given in Table 3. The Lifshitz–Van der Waals values of all three filaments are very close to each other and the difference is not statistically significant (p > 0.05). There is an increase in the electron acceptor energy of the drug-loaded filaments and a decrease in the electron donor energy.

#### 3.3.4. Nano-Level Gaps Analysis

PALS was used to detect nano-level gaps in filament structure, and the results are presented in Table 4. Gaps in the filament structure are thought to directly affect the filaments’ biodegradation time and drug release profile [29,31]. With the addition of CBP and PCX into the filament structure, there is an increase in nanogaps. When the structure of PCX is compared to CBP, it is seen that the nanogaps increase slightly because PCX is a larger molecule than CBP. Incorporating CD inclusion complexes into the structure and their insertion between PCL chains reduces the frequent stacking of PCL chains, which leads to an increase in nano-sized cavities. When the data obtained as a result of the mechanical properties and the PALS data are evaluated together, it can be said that this porous structure formed in the filaments does not seriously affect the mechanical durability of the filament.

#### 3.3.5. Theoretical and Analytical Drug Amount

**Figure 8:**
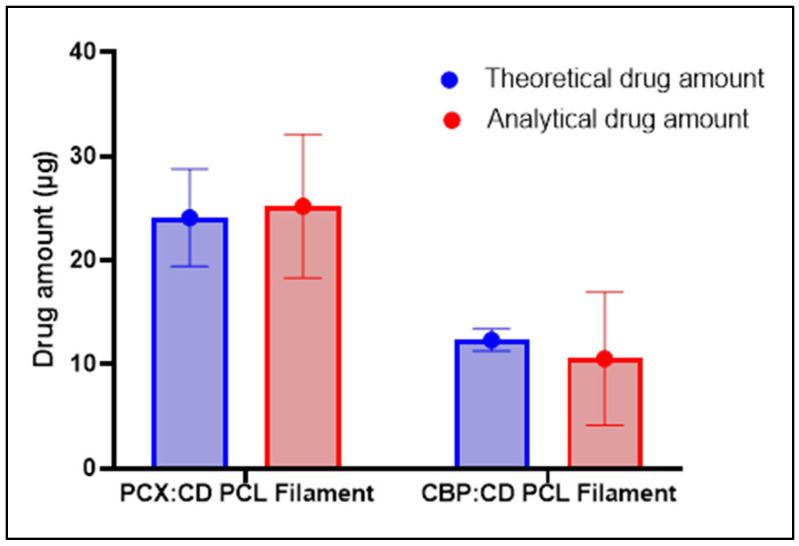
Analytical (red) and Theoretical Drug (blue) amount of PCX:CD PCL Filament and CBP:CD PCL Filament.

One centimeter samples were taken from each filament and weighed with a precision balance to calculate the theoretical amount of drug in each filament. In calculating this value, the ratio of the quantities of drugs and polymers used during preparation is considered. Accordingly, 1‰ of the total weight of the filament was calculated as PCX and 0.5‰ as CBP. In the calculation of analytical drug amounts, 1 cm samples were dissolved in a suitable solvent and drug amount were measured by the validated HPLC method. The theoretical and analytical drug amount for each drug is given comparatively in Figure 8. When these results are compared, it is seen that the filaments contain more than 85% of the drugs for both PCX and CBP. Thus, it was shown that the stability for each drug was maintained and loaded into the filaments at a high rate.

#### 3.3.6. Drug Release Profile

**Figure 9:**
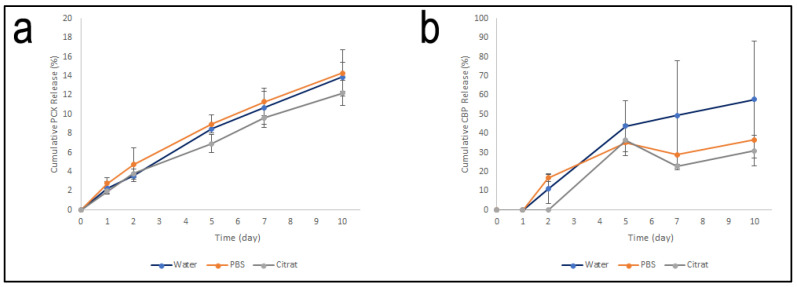
The cumulative drug release profile of (**a**) PCX:CD PCL Filament and (**b**) CBP:CD PCL Filament.

One centimeter samples were taken from the filaments prepared to determine the drug release profile in each filament. These samples were placed in release media and release profiles are given in Figure 9.

In PCX:CD filaments, no release was seen in the first 12 h for all 3-release media. It also showed a similar profile for 10 days in all release media. This result is an indication that PCL has similar properties in neutral (pH 7.4) and slightly acidic pH (pH 5.5). Figure 9a shows that only 15% of the PCX was released at the end of the 10 days. The fact that the release occurs so slowly leads to the idea that PCX is confined to the inner cavities of the filament as opposed to the filament surface and is released due to the degradation of PCL. Considering that the system is to be designed as an IUD by using a 3D printer, it is an indication that this system will allow the IUD to release PCX in a long-term and controlled manner.

CBP:CD filaments showed a faster release profile, unlike PCX:CD filaments (Figure 9b). In all three release media, CBP release was not observed during the first 24-hour period, followed by a controlled release for 10 days. The highest rate of release was seen in filaments in ultra-pure water. The solubility of CBP in water (≅40 mmol/L) [51] is quite high in contrast to PCX. The high solubility in the water is seen as one of the reasons why the release in the aquatic environment is faster. In addition, unlike PCX, the release of CBP was observed to occur independently of the degradation of PCL. CBP:CD inclusion complex are thought to concentrate on the surface of the filament and become free with the effect of the release medium.

The main mechanism for the sustained release of both drugs is thought to be related to the hydrolytic degradation of PCL. Due to the hydrolytic degradation of ester bonds of PCL, the DRUG:CD inclusion complex is thought to be released. Considering that the degradation of PCL is completed in 2–3 years, the sustained release of the drug is also due to this.

### 3.4. In Vitro Cell Culture Studies

**Figure 10:**
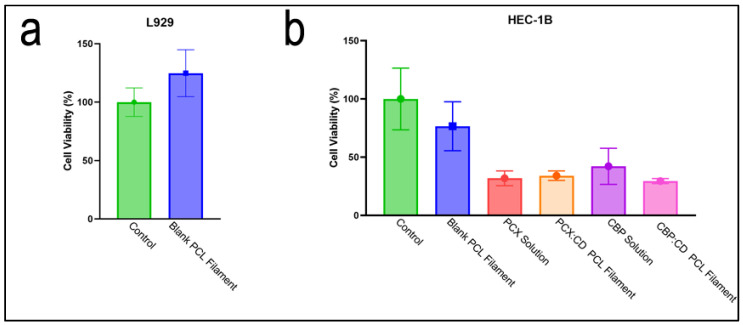
Cell viability of (**a**) drug-free filament on L929 mouse fibroblast and (**b**) drug-free filament, drug-loaded filament and drug solution on HEC-1B human endometrial adenocarcinoma cell line.

The cytotoxic effect of blank PCL filaments was examined in the L929 mouse fibroblast cell line to show that drug-free filaments do not have any toxic effects on healthy cells. As a result of this study, it was seen that filaments did not show any statistically significant effect on cell viability compared to the control group (Figure 10a). This result is an indication that drug-free filaments are safe and do not have any toxic effects on healthy cells.

The effect of drug-loaded filaments and drug solutions on cancer cells has been shown in cell line HEC-1B human endometrial adenocarcinoma cell line to show that drug-loaded filaments are at least as effective as drug solutions on cancer (Figure 10b). In addition, the cytotoxic effect of drug-free filaments on cancer cells was investigated in order to show that this anticancer effect is drug-induced and not related to the toxicity of PCL. As a result of this study, drug-loaded filaments have been shown to have a similar anticancer effect with drug solutions containing an equal drug amount to the first three days of drug release from the filament. When the effect of drug solutions and drug-loaded filaments on cell viability was compared, it was seen that the difference was not statistically significant. The cell culture result shows that the drugs maintain their effectiveness during the preparation steps and are successfully loaded into the filaments.

**Figure 11:**
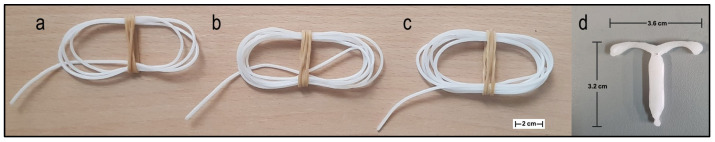
Images (**a**) Blank PCL Filament, (**b**) PCX:CD PCL Filament, (**c**) CBP:CD PCL Filament, and (**d**) 3D printed IUD.

The image of blank and drug-loaded filament optimized as a result of all these characterization studies and the prototype of the 3D printed IUD is given in Figure 11. As can be seen in the figure, filaments and 3D-printed IUDs using these filaments could be obtained successfully.

## 4. Conclusions

The drug-loaded filament makes it possible to manufacture a stable and precise 3D printed dosage form or drug-eluting device using the FDM technique, and therefore, is critical. With this study, a detailed characterization and optimization of filaments, which will be suitable for FDM 3D printers and allow the production of personalized IUDs or implants in cancer treatment was carried out. MβCD inclusion complexes were prepared with two different anticancer drugs, thus increasing the solubility and thermal properties of the drugs, and ensuring that they remained stable throughout the filament preparation processes. It has been shown that the PCL (M_n_ = 80,000) is a highly suitable polymer for developing filaments containing drugs for this purpose. The addition of Drug:CD inclusion complexes do not have an adverse effect but rather improves the filament’s mechanical properties. When the drug amounts in the filaments were compared with the theoretical amounts, it was shown that a success rate of over 85% was achieved for both drugs with high stability. Cell culture studies also confirm that drug-loaded filaments have at least as much cytotoxic effect as drug solutions. In addition, PCL filaments do not show any cytotoxic effects in healthy cells. Therefore, it can be said that PCL filaments are highly safe for implant or drug preparation. Drug release studies have also shown that preparing IUDs with these filaments will contribute to the treatment of uterine cancer by releasing PCX and CBP.

As a result, PCL filaments containing drug:CD inclusion complex are well suited for use with FDM 3D printers. Preparing these filaments with anticancer drugs will provide a great convenience in the manufacturing of personalized implants or IUDs.

## Figures and Tables

**Table 1:** Shore D and MFI values of PCL derivatives.

	Shore D	MFI (g/10 min)
PCL10 (M_n =_ 10,000)	51.0 ± 1.0	265.2 ± 1.4
PCL45 (M_n =_ 45,000)	52.2 ± 0.8	15.4 ± 0.7
PCL80 (M_n =_ 80,000)	53.6 ± 0.5	5.7 ± 0.1

**Table 2:** Mechanical properties data of blank and drug-loaded filaments (*n* = 3).

	Blank PCL Filament	PCX:CD PCL Filament	CBP:CD PCL Filament
E_mod_ (MPa)	169.6 ± 17.8	144.3 ± 21.8	159.9 ± 11.7
F at 0.2% plastic strain (N)	11.3 ± 2.3	7.0 ± 0.7	8.4 ± 1.5
F_break_ (MPa)	29.2 ± 9.1	36.3 ± 0.5	33.4 ± 7.3
dL at break (%)	1234.8 ± 278.3	1440.2 ± 74.5	1314.0 ± 148.1

**Table 3:** Surface free energy data of blank and drug loaded filaments (*n* = 6).

Surface Free Energy of Filaments				
	γ_S_^LW^	γ_S_^+^	γ_S_^−^	γ_S_^Total^
Blank PCL Filament	25.02	0.41	25.43	50.9
PCX:CD PCL Filament	24.51	1.01	7.02	32.5
CBP:CD PCL Filament	24.81	0.66	17.52	43.0

**Table 4:** PALS analysis results of blank and drug-loaded filaments (*n* = 3).

	Gaps (nm)
Blank PCL Filament	0.325
PCX:CD PCL Filament	0.509
CBP:CD PCL Filament	0.492

## Data Availability

Not applicable.
